# Recent Advances and Future Directions in Syncope Management: A Comprehensive Narrative Review

**DOI:** 10.3390/jcm13030727

**Published:** 2024-01-26

**Authors:** Anna Maria Martone, Iris Parrini, Francesca Ciciarello, Vincenzo Galluzzo, Stefano Cacciatore, Claudia Massaro, Rossella Giordano, Tommaso Giani, Giovanni Landi, Michele Massimo Gulizia, Furio Colivicchi, Domenico Gabrielli, Fabrizio Oliva, Giuseppe Zuccalà

**Affiliations:** 1 Fondazione Policlinico Universitario “A. Gemelli” IRCCS, L.go A. Gemelli 8, 00168 Rome, Italy; annamaria.martone@policlinicogemelli.it (A.M.M.); francesca.ciciarello@policlinicogemelli.it (F.C.); vincenzo.galluzzo@policlinicogemelli.it (V.G.); giovanni.landi@policlinicogemelli.it (G.L.); giuseppe.zuccala@unicatt.it (G.Z.); 2 Department of Geriatrics, Orthopedics, and Rheumatology, Università Cattolica del Sacro Cuore, L.go F. Vito 1, 00168 Rome, Italy; claudia.massaro02@icatt.it (C.M.); rossella.giordano01@icatt.it (R.G.); tommaso.giani01@icatt.it (T.G.); 3 Department of Cardiology, Mauriziano Hospital, Largo Filippo Turati, 62, 10128 Turin, Italy; 4 Division of Cardiology, Garibaldi–Nesima Hospital, Via Palermo, 636, 95122 Catania, Italy; michele.gulizia60@gmail.com; 5 Division of Cardiology, San Filippo Neri Hospital-ASL Roma 1, Via Giovanni Martinotti, 20, 00135 Rome, Italy; furio.colivicchi@aslroma1.it; 6 Department of Cardio-Thoracic and Vascular Medicine and Surgery, Division of Cardiology, S. Camillo-Forlanini Hospital, Circonvallazione Gianicolense, 87, 00152 Rome, Italy; dgabrielli@scamilloforlanini.rm.it; 7 “A. De Gasperis” Cardiovascular Department, Division of Cardiology, ASST Grande Ospedale Metropolitano Niguarda, Piazza dell’Ospedale Maggiore, 3, 20162 Milan, Italy; fabrizio.oliva@ospedaleniguarda.it

**Keywords:** syncope, transient loss of consciousness, vasovagal syncope, reflex syncope, orthostatic hypotension, cardiac syncope, electrophysiological study, tilt testing, syncope unit, falls

## Abstract

Syncope is a highly prevalent clinical condition characterized by a rapid, complete, and brief loss of consciousness, followed by full recovery caused by cerebral hypoperfusion. This symptom carries significance, as its potential underlying causes may involve the heart, blood pressure, or brain, leading to a spectrum of consequences, from sudden death to compromised quality of life. Various factors contribute to syncope, and adhering to a precise diagnostic pathway can enhance diagnostic accuracy and treatment effectiveness. A standardized initial assessment, risk stratification, and appropriate test identification facilitate determining the underlying cause in the majority of cases. New technologies, including artificial intelligence and smart devices, may have the potential to reshape syncope management into a proactive, personalized, and data-centric model, ultimately enhancing patient outcomes and quality of life. This review addresses key aspects of syncope management, including pathogenesis, current diagnostic testing options, treatments, and considerations in the geriatric population.

## 1. Introduction

Syncope is a condition characterized by a sudden and temporary loss of consciousness (TLOC) caused by a decrease in blood flow to the brain. It is marked by a rapid onset, brief duration (a few seconds up to a minute), and spontaneous full recovery [1]. The incidence and prevalence of syncope are similar in men and women [2], with the lifetime cumulative incidence being over 35% [3]. Syncope accounts for 0.6–3% of all the emergency department (ED) visits globally [4,5]. Approximately 50% of them are admitted; however, there are significant differences, ranging from 12% to 86% [1]. Epidemiological evidence reveals that the incidence has a first peak between the second and the third decade of life and a second peak after the age of 80 [2,3,6,7,8]. However, although syncope is such a common condition, its management remains challenging. According to a recent survey, despite guidelines being issued by major scientific societies [1,9], in 43.6% of cases, ED management of syncope is not standardized [10]. Standardization, as well as a proper history recording on admission, is crucial for an effective differential diagnosis with other conditions presenting as TLOC and the identification of patients with potentially life-threatening admissions [10,11]. 

In this review, we highlight the main issues related to the management of syncope. We discuss its pathogenesis, current diagnostic and treatment options, as well as relevant factors in the geriatric population. We finally highlight the limitations of current knowledge and address perspectives on the future.

## 2. The Baroreceptor Reflex

**Figure 1:**
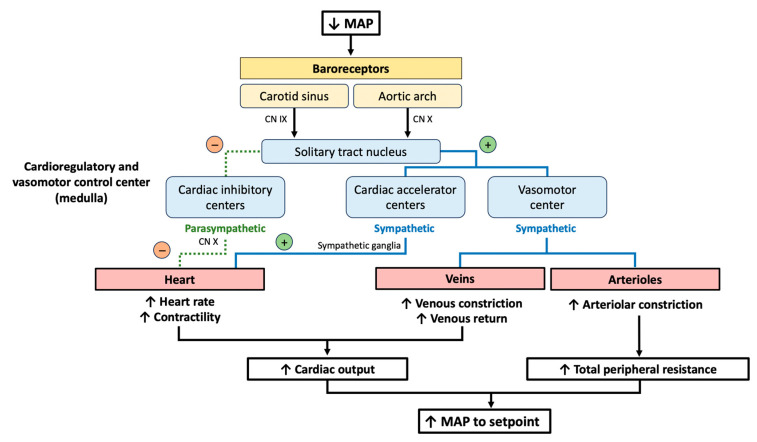
Physiology of the baroreceptor reflex. Abbreviations: CN: cranial nerve; MAP: mean arterial pressure.

The baroreceptor reflex regulates the short-term control of blood pressure (BP) during postural changes and the rest-to-exercise transitions by operating as a negative feedback control system (Figure 1) [12]. The baroreceptors located in the carotid sinus and in the aortic arch detect variations in the mean arterial pressure (MAP) and send information to the cardioregulatory and vasomotor centers of the medulla. MAP is influenced by cardiac output and systemic vascular resistance. In the case of a reduction in the MAP, the firing rate of the baroreceptors to the solitary tract nucleus (NTS) decreases. The NTS inhibits the nucleus ambiguus, thus reducing parasympathetic activity, and activates the cardiac accelerator and vasomotor centers, stimulating sympathetic activity. In the heart, the inhibition of the nucleus ambiguus reduces the parasympathetic tone on the sinoatrial node, increasing heart rate (HR). Concurrently, sympathetic activity increases heart contractility and stimulates the constriction of the venous wall, enhancing venous return (pre-load). In the arterioles, the sympathetic nervous system stimulates vasoconstriction. Through this mechanism, the baroreceptor reflex influences both cardiac output and total peripheral resistance to restore the MAP to the original setpoint [12].

Advancing age is a prominent risk factor for baroreflex dysfunction. Indeed, BP variability has been addressed as a key marker of aging [13,14]. In older adults, several factors compromise optimal BP control, including arterial stiffness [15,16], endothelial dysfunction [17,18], atherosclerosis [19,20], chronic inflammation and oxidative stress [21], decreased baroreflex sensitivity [22], and medications [23,24]. Other conditions decrease baroreflex sensitivity through primary or secondary dysautonomia. Primary causes of dysautonomia include pure atrophy, multisystem atrophy, Parkinson’s disease, and Lewy body dementia [25]. Secondary dysautonomia is caused by diabetes [26], amyloidosis [27], uremia [28], metabolic syndrome [29], heart failure [30], or other cardiovascular and non-cardiovascular diseases [31,32], as well as drugs causing sympathetic inhibition (e.g., antipsychotics via α1 and d2, antidepressants via antimuscarinic activity), vasodilation, cardio-inhibitory effects, or volume depletion (diuretics) [33]. Both acute SARS-CoV-2 infection and its post-acute sequelae are characterized by autonomic dysfunction and an altered mechanism of blood pressure variability and may manifest as syncope [34,35]. Interestingly, physical activity has shown benefits for baroreflex function in older adults [36]. Moreover, emerging evidence addresses the role of SGLT2 inhibitors in reducing cardiac autonomic neuropathy dysfunction [37,38,39].

## 3. Etiological Classification 

Syncope can be classified into non-cardiac syncope (including neurally mediated or reflex syncope and orthostatic syncope) and cardiac syncope, depending on its pathogenesis [1]. Identifying the underlying cause of syncope is essential for risk stratification. While neurally mediated syncope is usually benign, cardiac syncope is more often associated with worse outcomes, including death [1]. While syncopal events occurring in the first decades of life are mostly isolated with a lower risk of adverse outcomes that may benefit from lifestyle-adjustment therapy, syncopal events occurring in older adults are mostly associated with an underlying disease and usually require accurate evaluation and mechanism-specific therapy [8].

### 3.1. Non-Cardiac Syncope

According to the 2018 European Society of Cardiology (ESC) guidelines, non-cardiac syncope can be classified into reflex (or neurally mediated) syncope and orthostatic syncope [1].

Reflex syncope is the most common type and often has a benign course. It can be due to different causes that lead to an exaggerated baroreflex response, resulting in peripheral vasodilatation and/or bradycardia, with a consequent BP drop and cerebral hypoperfusion (Table 1) [1]. 

**Figure 2:**
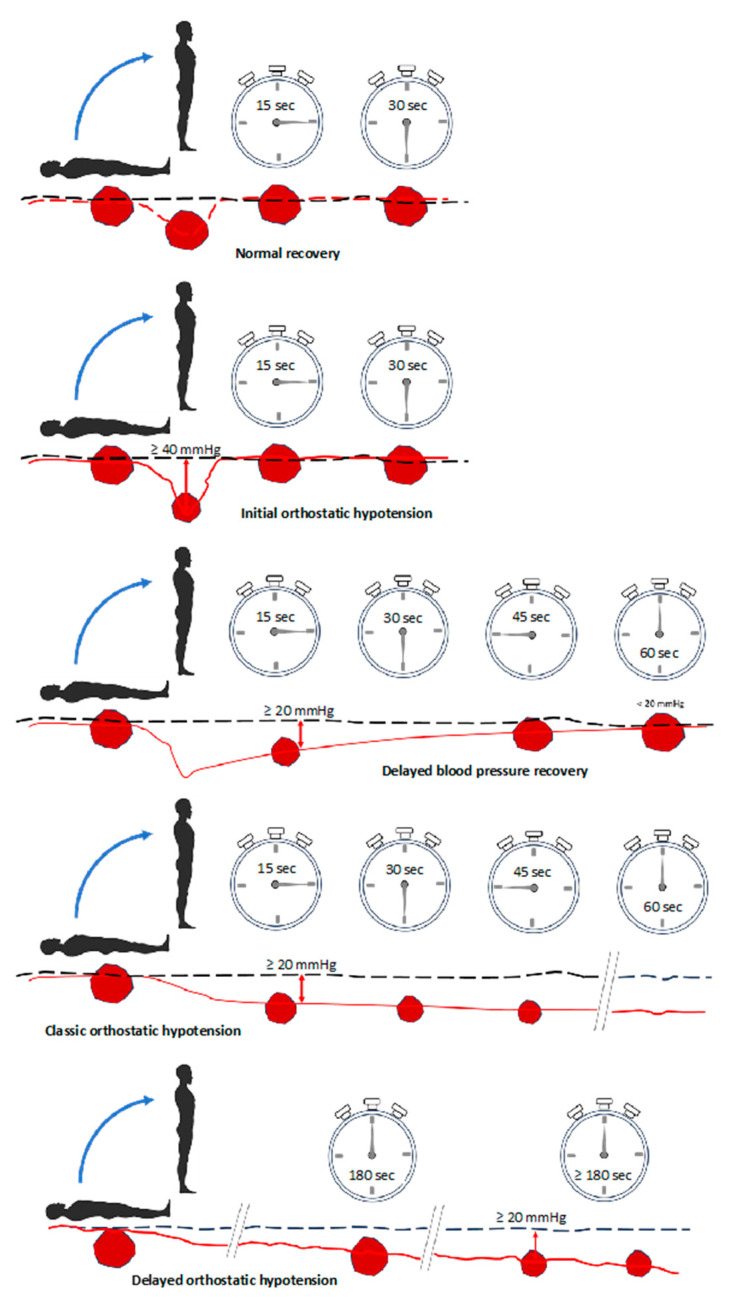
Continuous blood pressure curves showing normal recovery and the diagnostic criteria for initial delayed orthostatic hypotension. A decrease in the size of the red spots indicates a drop in blood pressure.

Orthostatic syncope refers to a TLOC resulting from a postural decrease in BP. It can occur with or without premonitory symptoms (e.g., dizziness, asthenia, fatigue, palpitations, sweating, visual and auditory disturbances, and neck pain). The causes of orthostatic hypotension (OH) include volume depletion (e.g., vomiting, diarrhea, hemorrhage, and Addison’s disease), dysautonomia, and peripheral venous pooling (e.g., exercise, post-prandial, or after prolonged bed rest), which may exacerbate drops in systolic BP (SBP) [1]. ESC guidelines identify five subtypes of OH (Figure 2) [1]. Initial OH is defined as a drop in SBP higher than 40 mmHg and/or a DBP higher than 20 mmHg occurring within the first 15 s. It is more common in older adults, and it is often drug-induced. Classic OH is defined as a decrease in SBP > 20 mmHg and/or DBP > 10 mmHg or a sustained SBP below 90 mmHg after over 3 min of active standing or tilting. Delayed OH occurs after 3 min of active standing or tilting. It is potentially caused by decreased pre-load and low cardiac output and differentiates from reflex syncope due to the absence of bradycardia. Vasovagal syncope triggered by orthostatism is more common in women with orthostatic intolerance. Postural orthostatic tachycardia syndrome (POTS) is defined by an increase in heart rate higher than 30 bpm or above 120 bpm within the first 10 min of active standing or tilting. It is due to an inappropriate increase in heart rate without a concomitant decrease in blood pressure. POTS mostly affects young women, individuals suffering from an infection or recent trauma, and those with joint hypermobility syndrome [1,40].

Distinguishing between the cardiac and non-cardiac causes of syncope is critical to performing proper risk stratification and guiding diagnostic and therapeutic management. While the 2018 ESC guidelines point out the existence of a “low BP phenotype” for vasovagal syncope, a recent multicohort cross-sectional study by Brignole et al. suggested that they may actually have different cardiovascular hemodynamics [41]. In particular, the hemodynamic profile of those identified as being the “low BP phenotype” may be characterized by a lower SBP and pulse pressure and, therefore, a reduced pre-load and lower stroke volume, and a higher HR and diastolic BP (DPB) as a likely activation of compensatory mechanisms. Therefore, recent evidence has focused on phenotypes rather than on classical etiological classification, such as the hypotensive phenotype, characterized by prevailing hypotension or vasodepression, and the bradycardic phenotype, with prevailing cardioinhibition [42,43].

### 3.2. Cardiac Syncope

Cardiac syncope can be associated with a variety of causes, including arrhythmias (bradyarrhythmia/tachyarrhythmia), heart disease (e.g., aortic stenosis, hypertrophic cardiomyopathy, cardiac tamponade, pericarditis, myocardial infarction, atrial myxoma, and prosthetic valve dysfunction), congenital anomalies of the coronary arteries, and pulmonary embolism [1,44]. While syncope globally has low morbidity and mortality, cardiac syncope is a red flag for sudden cardiac death, with its 1-year mortality reaching up to 30% [45].

## 4. Diagnostic Approach

**Figure 3:**
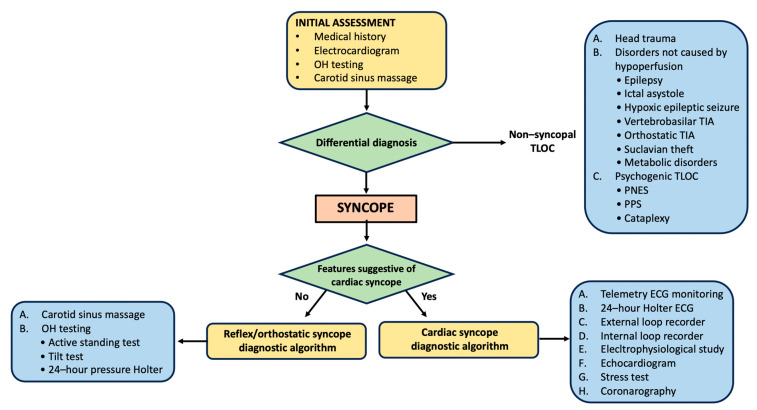
Diagnostic approach to syncope. Abbreviations: ECG: electrocardiogram; PNES: psychogenic non-epileptic seizure; PPS: psychogenic pseudoscope; OH: orthostatic hypotension; TIA: transient ischemic attack; TLOC: temporary loss of consciousness.

Studies have highlighted significant differences in managing syncope between different centers [46], with poor adherence to internationally established guidelines [11]. A lack of standardization is associated with higher costs due to unnecessary hospitalizations, especially in older adults [47], as well as unnecessary exams [48]. Further efforts from both research and governance are needed to provide more precise methods for identifying individuals at increased risk of complications, as well as to establish procedures that can optimize treatment for each patient [49,50,51]. An effective diagnostic approach for syncope requires four steps: (1) differentiating syncope from other forms of non-syncopal TLOC; (2) prognostic stratification; (3) differentiating between cardiac and non-cardiac syncope and following the appropriate diagnostic algorithm; (4) identifying the “red flags” that indicate serious health conditions manifesting as syncope (Figure 3). 

### 4.1. Initial Assessment

**Figure 4:**
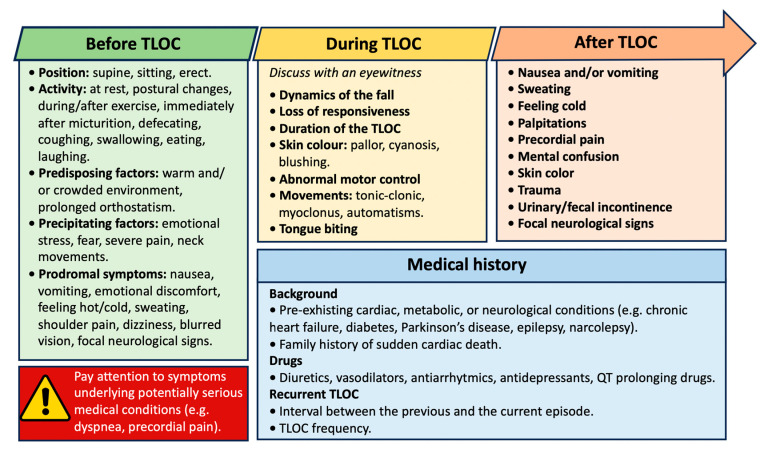
History recording in the initial assessment of syncope. Abbreviations: TLOC: temporary loss of consciousness.

The initial assessment should differentiate syncope from other forms of non-syncopal TLOC. Reconstructing the fall dynamics with the patient or an eyewitness, identifying indications and symptoms that happened before, during, or after the fall, and an accurate pathological, pharmacological, and family history are all required for a proper initial assessment. ECG, OH testing, and carotid sinus massage (CSM) are mandatory in the early stages (Figure 4) [52].

### 4.2. Differential Diagnoses

Non-syncopal TLOC includes both traumatic (e.g., subarachnoid hemorrhage) and non-traumatic conditions. Among non-traumatic non-syncopal TLOC, epilepsy is the most common condition requiring a differential diagnosis (Table 2). Albeit rare (<60% of cases), myoclonia occurring in syncope may be confused with epileptic tonic-clonic contractions; however, they are often localized to one limb, are asynchronous and asymmetrical, and have a short duration. Occasionally, epilepsy and syncope may trigger each other. In 90% of cases, epileptic seizures are associated with changes in heart rhythm. Prolonged (>8 s) bradycardia caused by temporal lobe epilepsy may trigger the vagus nerve and cause syncope (ictal asystole). The cessation of cortical activity due to global cerebral hypoperfusion stops the convulsive attack. Treatment consists of antiepileptic drugs and PM implantation. On the other hand, a syncopal epileptic seizure may occur as a consequence of hypoxia. In this case, the epileptic seizure lasts longer than anoxia-dependent irritation [52]. 

Other causes of TLOC include vertebrobasilar transient ischemic attacks (TIAs), orthostatic TIAs, and subclavian steal syndrome. Vertebrobasilar TIAs usually last a few minutes and are characterized by a loss of consciousness accompanied by ataxia, vertigo, diplopia, nystagmus, dysarthria, and oropharyngeal dysfunction. Orthostatic TIAs occur as a combination of multiple cerebral artery stenoses and OH due to repetitive TIAs occurring in the orthostatic position. In subclavian steal syndrome, syncope is accompanied by neurological signs [52]. Metabolic disorders, such as hypoglycemia, hyperventilation with hypocapnia, and intoxication, may cause non-syncopal TLOC [52].

Finally, loss of consciousness may be merely apparent, referred to as psychogenic loss of consciousness. This condition can be categorized into two distinct types, namely a non-epileptic psychogenic convulsive seizure (PNES) and psychogenic pseudosyncope (PPS) [52]. In a PNES, patients present massive limb movements that strongly resemble an epileptic seizure. An electroencephalogram (EEG) and video-EEG may help to discriminate between psychogenic and organic seizures. Although it is classified as a psychiatric disorder rather than a true TLOC, PPS shares significant similarities with reflex syncope. It can be preceded by pre-syncopal prodromal symptoms, such as vision changes, frostbite, sweating, and shortness of breath, and its causative factors include prolonged orthostatic position, a crowded environment, and psychological tension. Differently from syncope, however, PPS may last up to 15–30 min or even hours. During the attacks, the patient’s eyes are shut, and they exhibit no response to physical touch or verbal stimulation. However, individuals with PPS show signs that are inconsistent with a loss of consciousness, such as fluttering eyelids, swallowing, retained muscle tone, normal limb movement, and aversion to opening the eyes. The gold standard for diagnosing PPS is video documentation during a tilt test (preferably with an EEG) with a normal BP and HR. Once diagnosed, it is necessary to reassure the patient that it is not a “voluntary” condition. Acceptance of the diagnosis is crucial to refer the patient for cognitive behavioral therapy and allow an immediate reduction in the frequency of the episodes [52]. Assessing emotional and psychogenic factors should be considered in the management of syncope. France et al. studied the determinants of fear and anxiety as triggers of vasovagal syncope in the context of blood donation and identified individuals who were more at risk of a vasovagal reaction following a venipuncture [53]. O’Hare et al. identified childhood trauma as a possible determinant of life-long vasovagal tendency [54]. A study of 162 individuals with positive tilt tests for vasovagal syncope, which were matched with 162 healthy subjects found that patients who experienced syncope had higher scores in their persistence temperament and self-transcendence character traits [55]. A further study highlighted a higher prevalence of depression and anxiety in patients with vasovagal syncope [56,57]. Those findings imply the potential utility of psychological assessment when treating patients with refractory vasovagal syncope.

Other conditions that may mimic TLOC are cataplexy and drop attacks. Cataplexy is a sudden and transient episode of muscle weakness accompanied by full conscious awareness, typically triggered by emotions (e.g., laughter) without amnesia. The term drop attacks refers to sudden falls without warning signs and/or symptoms and may be used to describe an atonic seizure, Meniere’s disease, or a specific syndrome of unknown etiology [52].

### 4.3. Risk Stratification

Once the differential diagnosis between syncopal and non-syncopal TLOC has been ruled out, the diagnostic algorithm requires the identification of patients at high risk, neither high nor low risk, and low risk (Table 3). High risk requires urgent hospitalization, moderate risk demands a fast track to the syncope unit (in-hospital or as an outpatient), and low risk necessitates outpatient evaluation and may benefit from reassurance or counseling [58]. Numerous syncope prediction tools have been developed through the combination of different clinical variables to improve risk stratification (e.g., the Martin-Kapoor score [59], the osservatorio epidemiologico sulla sincope nel Lazio (OESIL) risk score [60], the San Francesco syncope rule [61], the FAINT score [62], the Basel IX ECG ALERT-CS tool [63]), and the evaluation of guidelines in syncope study (EGSYS) score [64]. However, the main limitation in implementing a prognostic score in clinical practice was the lack of reproducibility and heterogeneity in study designs, populations, and outcomes [58]. In the real world, those scores did not perform better than clinical judgement; therefore, both European and American guidelines recommend against their use as a unique criterion for risk stratification [1,9]. The prognosis of patients with syncope strictly depends on the etiology [65]. The one-year mortality of cardiac syncope is significantly higher (18–33%) than non-cardiac subtypes (3.4%) [66]. The subsequent choice of a diagnostic test should be guided by pre-test probability. In 90% of cases, an etiological diagnosis can be decided upon with only two more tests in addition to the initial assessment [1]. 

#### 4.3.1. Diagnosis of Non-Cardiac Syncope

CSM and orthostatic challenges (active standing and tilt test) are complementary tests for the diagnosis of non-cardiac syncope. While CSM and the tilt test showed a minimal overlap in bradycardic patients (3%), the tilt test was able to correctly identify 98% of patients with hypotensive syncope. Therefore, the authors concluded that CSM and orthostatic challenge are indicated for identifying those patients with a bradycardic phenotype; however, CSM has limited utility for the hypotensive phenotype [67].

CSM is indicated in patients over 40 years of age with a suspicion of reflex syncope. Asystole > 3 s and/or a fall in BP > 50 mmHg after CSM suggests carotid sinus syndrome [68]. If CSM causes pure asystole, the possibility of a marked but hidden concomitant vasodepressor response should be considered. In that case, the intravenous administration of 0.02 mg/kg atropine prevents reflex asystole, allowing the unmasking of any concomitant vasodepressor response. A history of TIA or stroke occurring in the previous six months and/or stenosis higher than 70% in the common or internal carotid arteries are contraindications for performing CSM [69]. Other contraindications include a recent myocardial infarction (<8 weeks) and a history of ventricular arrhythmias. The main complications of CSM are TIAs and ischemic events; however, they are rare (0–24%) [69]. CSM is always reproducible in individuals with severe cardioinhibitory conditions who are candidates for PM implantation. A cardioinhibitory response to carotid sinus massage that reproduces symptoms appears to be predictive of spontaneous asystole [65,70,71,72,73]. Orthostatic challenge tests include the active standing test, tilt test, Valsalva maneuver, deep breathing test, and 24-h Holter BP test (Table 4) [74]. 

The active standing test allows for the diagnosis of initial OH and classic OH. A symptomatic systolic fall of 20 mmHg or a diastolic fall of 10 mmHg from the clinostat values or an absolute BP value below 90 mmHg indicates OH. However, OH syncope remains a suspected diagnosis if the symptoms are not reproduced. The tilt test identifies patients with hypotension susceptibility (e.g., suspected vasovagal syncope with an atypical presentation, recurrent syncope without heart disease, syncope with heart disease and negative tests for cardiac syncope, older adults with recurrent unexplained falls, and syncope without prodromes and with retrograde amnesia). The tilt test mimics the clinical situation of a vasovagal syncope induced via prolonged orthostatism or in patients susceptible to a vasovagal response. Passive orthostatism induces a vigorous “empty heart” myocardial contraction, which leads to an inappropriate activation of ventricular mechanoreceptors and subsequent paradoxical vasodilation and cardiac inhibition. An asystolic pause may precede or coincide with TLOC, and this has important clinical implications for PM implantation. However, while a cardioinhibitory response in the tilt test is highly predictive of asystolic syncope, the presence of a vasodepressor or a mixed or even negative response does not exclude the presence of asystole during spontaneous syncope. The tilt test has low specificity and sensitivity in patients with syncope of an uncertain cause (30–36%) [75]. Studies have reported a positive test in 51–56% of patients with atypical clinical features suggesting reflex syncope, 30–36% of patients with unexplained syncope, and 45–47% of patients with arrhythmic cardiac syncope [76]. In the latter group, a positive tilt test reveals orthostatic stress susceptibility, which plays a role in determining syncope regardless of etiology and mechanism. The alternative hemodynamic profile of those identified as having the low BP phenotype, however, [41] may pair the concept of hypotension susceptibility. A tilt test yielding an abnormal result does not definitively diagnose vasovagal syncope but rather indicates a predisposition to hypotension that becomes evident while standing upright. Therefore, any predisposition towards OH may become activated in the upright position and result in vasovagal syncope [77]. One more relevant issue in the diagnosis of vasovagal syncope is the quantification of vasodepression and cardioinhibition and their contribution to BP [77]. For this purpose, Van Dijk and colleagues proposed the log-ratio method to assess the relationships between vasodepression, cardioinhibition, and peripheral resistance on BP [78]. According to their study conducted in a cohort of 163 patients, cardioinhibition occurred in 91% of patients after a mean time of 58 s from TLOC, and it manifested as a reduction in the stroke volume. While peripheral resistance was not impacted by cardioinhibition, the reduction in the stroke volume negatively impacted BP and produced an ineffective corrective increase in HR, which was unable to compensate for the decline in BP [78]. In a subsequent study, the authors highlighted that aging had a significative impact on cardioinhibition and vasodilation, as it was associated with a diminished increase in HR and a faster BP decline [79]. Therefore, the authors questioned the utility of pacing in older patients with vasovagal syncope.

Baseline tests such as the Valsalva maneuver, deep breathing test, and 24-hour ambulatory blood pressure monitoring (ABPM) can be performed to determine if the underlying cause of OH is dysautonomia. Recent evidence addresses ABPM as a potentially useful diagnostic test for reflex syncope. According to the SynABPM 1 study, reflex syncope patients showed a higher frequency of SBP drops than healthy controls [80]. Therefore, the authors proposed the novel cut-off values of one episode of daytime SBP < 90 mmHg or two episodes of daytime SBP < 90 mmHg if the mean 24-h SBP is below 125 mmHg to identify reflex syncope with hypotensive susceptibility [80].

#### 4.3.2. Diagnosis of Cardiac Syncope

In cardiac syncope, the primary event is a marked reduction in cardiac output due to arrhythmia, structural heart disease, or pulmonary embolism. 

Presyncope, symptomatic arrhythmias without diagnostic ECG criteria, and sinus bradycardia without syncope are necessary but not sufficient to identify cardiac syncope. On the other hand, significant tachy- or brady-arrhythmias (asystole for more than 3 s, second-degree atrioventricular (AV) block Mobitz II, third-degree AV block, rapid and prolonged supraventricular tachycardia, or ventricular tachycardia), even without documented syncopal events, are considered diagnostic findings. Diagnostic tests for cardiac syncope include tools to identify intermittent tachy- or brady-arrhythmias (telemetry ECG monitoring, 24-h Holter ECG, and an external/internal event recorder) and electrophysiological (electrophysiological study, EPS) or morphological (echocardiography) substrate, and exercise testing (Table 5) [1,44].

Despite its low diagnostic power (1.9–17.6%), telemetry ECG monitoring is useful in the first 72 h of a TLOC event in high-risk patients. The 24-h Holter ECG is indicated in patients with very frequent syncope or presyncope (>1/week); otherwise, the diagnostic power is 1–2% [81]. For sporadic episodes, event recorders are more useful [82]. An external loop recorder can be used for recurrent syncope (>1/month), while an internal loop recorder is indicated for indeterminate syncope in high-risk patients, low-risk patients with a high probability of recurrence within 36 months (≥3 episodes in the previous 2 years), and to determine the contribution of bradycardia to TLOC (and thus to indicate PM implantation) in patients with reflex syncope [1,44].

An electrophysiological study (EPS) is recommended to identify arrhythmic syncope in patients with a history of organic or acquired heart disease or/and the presence of abnormal findings on the ECG [83]. 

If a structural cardiac disease is suspected during the initial assessment, an echocardiography is useful for both diagnosis and prognostic stratification to assess the risk of sudden death. An ejection fraction (EF) < 35% is associated with a high risk of sudden death in 2 years; therefore, it is an indication of implantable cardiac defibrillator (ICD) implantation regardless of the cause of syncope [1,84].

Exercise testing is recommended for patients experiencing exertional syncope, aiding in a diagnosis when syncope is replicated during or immediately after physical activity in conjunction with ECG alterations (or when a second- or third-degree AV block manifests even without accompanying syncope). It is crucial to note that, in the absence of structural heart disease, exercise-induced syncope may be an expression of vasovagal vasodilation [1,85,86].

Coronary angiography does not contribute to syncope diagnosis; however, it proves beneficial in cases of heart disease with systolic dysfunction by evaluating the potential for myocardial revascularization, aiming to enhance the patients’ prognoses [1,87].

## 5. Treatment

The treatment of syncope aims to reduce syncopal recurrences; however, no treatment is completely effective in eliminating syncope episodes in the long term [88]. The underlying physiological mechanism of syncope influences the choice of treatment. For example, bradycardia is the most common cause of cardiac syncope, and it is treated with PM implantation. Nevertheless, the coexistence of hypotension reduces pacing efficacy [79,88]. On the other hand, the management of reflex or orthostatic syncope is more complex since specific therapies are less effective [89]. However, syncopal recurrences often spontaneously resolve after medical evaluation, even without any specific therapy [1]. 

Syncope tends to recur in less than 50% of patients within 1–2 years, particularly in cases of reflex and orthostatic hypotension syncope [1]. The underlying reason for this decrease is unknown. This implies that treatment may be delayed in low-risk conditions, and caution is warranted when interpreting the effectiveness of interventions in preventing syncope, as observational studies lacking a control group may be biased [1].

### 5.1. Treatment of Reflex Syncope

The goal of treating reflex syncope is to enhance the quality of life. Non-pharmacological approaches involve providing reassurance about the benign nature of syncope and encouraging behavioral measures to eliminate or reduce triggers. These measures include avoiding prolonged standing or sitting, rapid changes in position, hot or crowded environments, and engaging in moderate physical activity if tolerated, along with staying hydrated and consuming salted foods. Recognizing prodromes and adopting safe positions or physical counter-pressure maneuvers can significantly reduce syncope recurrence [90].

**Figure 5:**
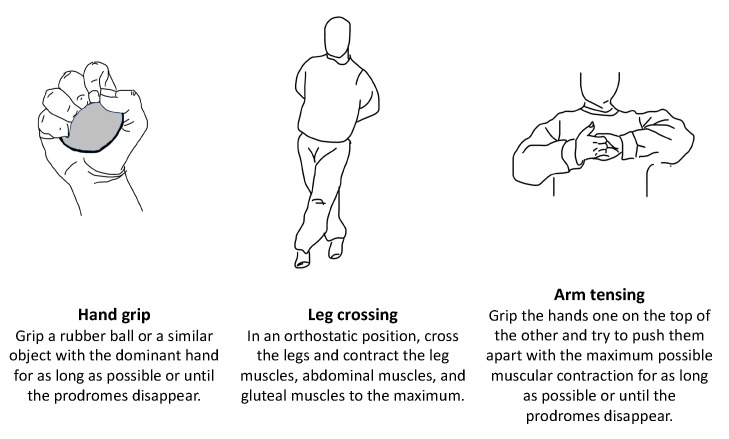
Physical counter-pressure maneuvers.

For cases of recurrent or disabling syncope, therapeutic strategies generally depend on age (younger than 40 years or older than 60 years). Treatment options include adjusting or withdrawing antihypertensive therapy, physical counter-pressure maneuvers (Figure 5, and tilt training [1,90]. Isometric muscle contractions increase cardiac outflow and BP and can potentially delay or prevent syncope events (Figure 5). Tilt training involves gradually increasing periods of orthostatism and may be effective in young, motivated patients with orthostatic stress-induced prodromes, although clear efficacy in clinical studies is not well-established [1,90].

Pharmacological measures are rarely employed [90,91]. Fludrocortisone increases renal sodium absorption, counteracting the physiological cascade triggering vasovagal reflex syncope. It is recommended at doses of 0.05–0.2 mg/day for young patients without comorbidities and consistently low BP values [1,90]. According to a recent meta-analysis, midodrine, an alpha-agonist acting as a vasoconstrictor, showed a reduction in the recurrence of vasovagal syncope (relative risk (RR) 0.55; 95% CI 0.35–0.85) [91]. Midodrine may be considered at doses of 2.5–10 mg three times a day for young patients with the low BP phenotype experiencing orthostatic-induced reflex vasovagal syncope [1,90].

In the case of a dominating cardioinhibitory response, the implantation of a dual-chamber PM should be considered. PM implantation is indicated in patients over 40 years old. One indication is the evidence on the internal loop recorder of symptomatic asystolic pauses >3 s or asymptomatic pauses >6 s due to sinus arrest or AV block. However, the presence of a concomitant reflex vasodilatory response compromises the efficacy of pacing, resulting in a higher probability of syncopal recurrence. Another indication is a positive tilt test for a cardioinhibitory response and recurrent syncope, although the evidence is controversial. The clinical presentation of syncope itself, along with the absence of concomitant vasodilation and hypotension, is crucial in selecting patients for implantation. Additional tests, such as an internal loop recorder (ILR), are necessary to document the exact mechanism of reflex syncope and determine the appropriateness of pacing. The tilt test, especially when assessing hypotensive susceptibility, is critical for identifying patients who may not effectively respond to pacing, as a positive tilt for hypotensive susceptibility is a strong predictor of PM ineffectiveness [1,92].

Cardioneuroablation (CNA) is gaining recognition for its effectiveness in treating vasovagal syncope. CNA refers to the process of precisely targeting and cauterizing the intrinsic epicardial ganglia within the heart. This results in a partial parasympathetic denervation, decreasing vagal tone on the cardiac fibers, reducing the incidence of bradyarrhythmia [93]. A meta-analysis by Vandenberk et al. on 465 patients aged 18–60 years affected by severely symptomatic recurrent reflex syncope with spontaneous or orthostatic challenge-induced asystole and without sinus node or AV node dysfunction was performed, involving 14 studies [94]. According to their results, the freedom-from-syncope (FFS) after CNA was 91.9% (95% confidence interval (CI) 88.1–94.6%; *p* = 0.376), with higher rates of FFS for left atrial ablation (94.0%; 95% CI 88.6–96.9%) and biatrial ablation (92.7%; 95% CI 86.8–96.1%) than right atrial ablation (81.5%; 95% CI 51.9–94.7%; *p* < 0.0001) [94]. A recent study conducted on 48 patients with recurrent syncope showed lower recurrence rates of syncope in the CNA group compared to the non-CNA group [95]. However, despite the promising results, multiple issues arose regarding the selection of potential patients, ideal ablation site, verification of the ablation effect, and long-term durability of the procedure [96,97]. For this reason, further clinical trials and real-world studies encompassing a substantial number of participants with a suitably long follow-up duration are needed.

### 5.2. Treatment of Orthostatic Hypotension and Orthostatic Intolerance

The management of orthostatic intolerance syndromes involves reassuring patients about the benign nature of syncope and adopting behavioral measures. Additionally, reducing antihypertensive therapy to achieve a target SBP of 140–150 mmHg is recommended [1,89]. Angiotensin-converting enzyme inhibitors, angiotensin receptor blockers, and calcium channel blockers are preferred over beta-blockers and thiazide diuretics. If symptoms persist, further interventions, such as physical counter-pressure maneuvers, elastic stockings, head-up tilt (sleeping with a pillow to prevent nocturnal polyuria, improve fluid distribution, and address nocturnal hypertension), and the use of midodrine and fludrocortisone should be considered [1,89]. Droxidopa, a central and peripheral alpha and beta agonist, was recently approved by the Food and Drugs Administration for the treatment of symptomatic neurogenic OH [98,99]. The main concerns, however, remain for the durability of its benefits [100,101]. Hauser et al. conducted a 12-week open-label study using droxidopa. The study found a notable improvement in the symptoms of neurogenic OH and daily activities compared to the baseline evaluation [102].

### 5.3. Treatment of Cardiac Syncope

In sick sinus syndrome, a documented correlation between an ECG and syncope warrants consideration for PM implantation. This recommendation extends to patients experiencing syncope with relief of asymptomatic pauses. PM implantation is also indicated for second- and third-degree AV blocks, bifascicular blocks with a positive EPS, or evidence on the ILR of a paroxysmal AV block. Notably, the guidelines emphasize the importance of an EPS and ILR, as a bifascicular block alone suggests a complete block in less than half of patients, with one-third receiving a final diagnosis of reflex syncope and another third remaining unexplained [1,44]. 

For patients with paroxysmal supraventricular tachycardia (nodal reentrant tachycardia, AV reentrant tachycardia, atrial flutter, and ectopic tachycardia) and syncope, catheter ablation is recommended as a first-line therapy. The role of antiarrhythmic therapy is limited to the bridging period before ablation or in cases of ablation failure [1,44].

ICD implantation is indicated for syncope due to ventricular tachycardia with an EF < 35% and for syncope in the presence of ischemic heart disease with induced ventricular tachycardia during electrophysiological study. It may be considered in patients with an EF > 35% and recurrent ventricular tachycardia syncope when catheter ablation or medical therapy proves unsuccessful or is not feasible [1,44].

ICD implantation is always recommended in patients with unexplained syncope and dilated cardiomyopathy with an EF < 35%. Consideration for ICD implantation should be provided to patients with unexplained long QT (LQT) syndrome and recurrent syncope (not meeting diagnostic criteria) despite beta-blocker therapy, particularly in LQT2 and LQT3 syndromes, as well as patients with a Brugada type 1 ECG pattern and unexplained syncope [1,44].

In individuals with HCM, the decision for ICD implantation depends on the identification of a high risk of sudden cardiac death using the European Society of Cardiology (ESC) HCM Risk-SCD score. For those at low risk, the implantation of an ILR should be considered instead of an ICD. Lastly, for patients with syncope related to structural heart disease, addressing the underlying cause is essential [1,44].

## 6. Management of Syncope in Older Adults

In older adults, the most common form of syncope is vasovagal, constituting 66.6% of the total, according to data from the Italian syncope study group [103]. The reflex form prevails in younger patients, while the dysautonomic form is more prevalent in older patients. Many older individuals experience prodromes, with the available data suggesting that symptoms like nausea, blurred vision, and sweating predict non-cardiac syncope, while dyspnea is predictive of cardiac syncope [104]. One-third of older patients with hip fractures have unexplained falls [105]. The diagnostic protocol proposed by the ESC can be applied even in those over 90 years old, providing an etiological diagnosis in 90% of patients [10,103]. Orthostatic testing, CSM, and tilt testing are well-tolerated, even in frail older individuals with cognitive impairment [106]. Mortality and recurrence rates increase with age and comorbidity [106].

It is strongly recommended to reduce or discontinue antihypertensive and psychotropic drugs in older patients with syncope, as the benefits outweigh the risks. The term “hypotensive TIA” denotes focal neurological signs of hypotension and syncope, even in patients without significant internal carotid artery stenosis. Misdiagnosis can lead to increased antihypertensive therapy and worsen syncopal recurrences [1]. 

Unexplained falls often indicate syncope and the same diagnostic algorithm for syncope should be followed. Patients with unexplained falls may deny loss of consciousness, showing retrograde amnesia of the event. In the absence of witnesses, distinguishing between syncope, epilepsy, TIA, and falls is challenging [1]. Multiple causes of syncope may coexist in older adults, necessitating a multifactorial and multidimensional evaluation [13]. Atrial fibrillation or aortic stenosis are often found but are not frequently the direct cause of syncope [107,108]. Failure to stabilize orthostatic blood pressure is present in more than 40% of individuals aged over 80 years, posing a risk factor for falls and syncope. Assessing cognitive and physical performance is recommended in older patients [1].

### Syncope Unit

The syncope unit involves a multidisciplinary collaboration of specialists (cardiologists, neurologists, geriatricians, and psychologists) responsible for the diagnosis and treatment of syncope [109]. The syncope unit should have and follow a protocol for the diagnosis and management of syncope with an equipped area including 12-lead ECG and 3-lead ECG monitoring, non-invasive beat-to-beat blood pressure monitoring with recording facilities for subsequent analyses, tilt tables, Holter monitors/external loop recorders, ILRs, follow-up of ILRs, 24-h blood pressure monitoring, and basic autonomic function tests. The unit should establish procedures for echocardiography, EPSs, exercise testing, and neuroimaging [1,109]. Van Zanten et al. proposed and validated the SU-19 score to quantify the adherence of syncope units to the best practices based on the evaluation of the structure, initial assessment, and diagnostic tests [110]. The syncope unit itself should plan to keep medical records that could also be used for research purposes. Quality indicators for the syncope unit include achieving a 20% reduction in unexplained syncope and less than 20% of low/intermediate-risk patients coming from the ED. The syncope unit should reduce the costs associated with incorrect procedures by 20% and improve outcomes (<5% readmission for syncope and recurrence at 1 year in <20% of implanted patients) [1,109].

## 7. Future Directions in the Management of Syncope: A Little Help from New Technologies?

The future of syncope management holds interesting prospects marked by advancements in health technologies. 

Artificial intelligence (AI) has shown promising results in analyzing vast datasets from multiple cohorts to identify patterns and highlighting specific patient subsets to improve risk stratification and management [111]. Implementing AI in syncope management may provide benefits in achieving a successful differential diagnosis between both syncopal and non-syncopal TLOCs and between different syncopal etiologies (event definition), risk stratification, and patient management, including the need for immediate intervention and hospitalization, downstream testing, and long-term monitoring strategies [112]. Recent studies have addressed AI as a potential means to overcome the limits of current risk stratification tools [113,114]. In a systematic review, Goh et al. expressed that machine learning algorithms hold 88.8% sensitivity, 81.5% specificity, and an overall accuracy of 85.8% for detecting syncope [114]. 

Working with AI-based models requires a vast availability of data. The sharing of health data across hospitals would undeniably provide benefits [115]. Nevertheless, this viewpoint still has certain limitations in terms of infrastructure and legislative restrictions [116,117]. The implementation of blockchain technology may overcome current issues in health data sharing between institutions [118], and its integration with AI may provide beneficial effects for the diagnosis, risk stratification, and management of many cardiovascular conditions, including syncope [119]. In addition, increasingly advanced wearable devices and sensors enable the identification of syncopal episodes, falls, and arrhythmic events, transmitting data for more timely emergency responses and with the possibility of helping with risk stratification based on the patient’s personal health record, vital parameter monitoring, and ECG parameters [120,121,122].

Embracing these innovative approaches holds the potential to transform syncope management into a more proactive, individualized, and data-driven paradigm, ultimately improving patient outcomes and quality of life.

## 8. Conclusions

While cardiac syncope is a symptom of an underlying disease, the traditional classification into reflex and orthostatic syncope is increasingly challenged by the identification of phenotypes with a tendency for bradycardia or hypotension. Correctly identifying these phenotypes is critical for setting up personalized and mechanism-specific diagnoses and treatments. A multidisciplinary approach proves advantageous in identifying and managing the most controversial and difficult cases, while in the future, new technologies could provide substantial help in identifying cases at higher risk for adverse outcomes. The ultimate goal is to maximize the harmonization of both the standardization and personalization of diagnostic and therapeutic procedures in order to reduce costs, minimize hospitalizations, and decrease morbidity.

## Figures and Tables

**Table 1:** Classification of reflex mediated syncope.

Subtype	Etiology	Features
Vasovagal	Emotional distress (e.g., pain and fear).	Usually occurs at a younger age
Situational	Coughing, sneezing, swallowing, defecation, and micturition after exercise	
Carotid sinus syndrome	Vagal hyperactivation due to accidental stimulation of carotid sinus baroreceptors	People ≥ 40 years old with increased baroreceptor sensitivity due to atherosclerosis and arterial stiffness
Atypical forms	No identifiable triggers	

**Table 2:** Differential diagnoses between syncope and epilepsy.

Syncope	Epilepsy
Before TLOC	
Prodromal symptoms	Rare triggers (e.g., exposure to flashing and flickering light in photosensitive epilepsy) Frequent auras.
During TLOC	
Myoclonia (rare, <60% of cases) Localized to one limb Asynchronous and asymmetrical Begin after TLOC Duration: 10–30 s Tongue biting is rare	Tonic-clonic contractions Localized to one hemisome or limb Synchronous and symmetrical Before or during the fall Duration: ≥1 min Frequent automatisms (e.g., chewing, lip smacking, mouth drooling, and biting the side wall of the tongue)
After TLOC	
Nausea, vomiting, and pallor Complete recovery of consciousness	Prolonged confusion and memory loss Muscle pain for hours or days due to muscle contractions
Other features	
Fecal/urine release, open eyes during TLOC, fatigue, and sleepiness are common in both conditions. Cyanotic face is common in epilepsy but rare in syncope	

Abbreviations: TLOC: temporary loss of consciousness.

**Table 3:** Characteristics of patients with high-risk syncope and low-risk syncope.

High-Risk Syncope
“Red flags” of a severe condition (e.g., chest pain, dyspnea, abdominal pain, headache, focal neurological signs, bleeding, severe anemia, and severe electrolyte disturbances) Structural heart disease Syncope in the supine position or during exercise Sudden onset of palpitations followed by syncope Family history of sudden death at a young age Absence of prodromes or short prodromes and structural heart disease/abnormal ECG Syncope in the sitting position and structural heart disease/abnormal ECG Unexplained BP < 90 mmHg Unknown systolic murmur Major ECG criteria for cardiac syncope: ○ Signs of acute ischemia; ○ Second-degree AV block Mobitz II or third-degree AV block; ○ Low ventricular response atrial fibrillation (<40 bpm), sinus bradycardia (<40 bpm), or repetitive sinoatrial block or pauses > 3 s and weakness at the rest state; ○ Bundle-branch block; ○ Intraventricular conduction disturbance; ○ Ventricular hypertrophy or Q waves (ischemic heart disease or cardiomyopathy); ○ Sustained/not sustained VT; ○ PM or ICD malfunction; ○ Brugada pattern; ○ QTc interval > 460 ms in repeated 12-lead ECG (long QT syndrome). Minor ECG criteria (suggesting syncope of cardiac origin only if the history is consistent with arrhythmic syncope): ○ Second-degree AV block Mobitz I and first-degree AV block with a long PR interval; ○ Bifascicular block; ○ Mild sinus bradycardia (40–50 bpm); ○ Unexplained or low ventricular rate response AF (40–50 bpm) without negative chronotropic drugs; ○ Signs of early repolarization; ○ Ventricular hypertrophy signs consistent with hypertrophic cardiomyopathy; ○ Paroxysmal VT and AF; ○ Short QT interval; ○ Atypical Brugada patterns; ○ Negative T-waves in right precordial leads; ○ Epsilon waves (right ventricular arrhythmogenic cardiomyopathy).
Low-risk syncope
Definite criteria for classic vasovagal syncope (e.g., emotional triggers or prolonged orthostatism associated with typical prodromal symptoms); Definite criteria for situational syncope (e.g., during/immediately after urination, coughing, defecation, swallowing, or laughing); Definite criteria for orthostatic syncope (e.g., documented OH after prolonged standing, temporal link with the beginning or changes in vasodilator therapy, TLOC preceded by pain radiating from the neck to the shoulders and trunk). ○ Definite criteria for reflex syncope (e.g., absence of cardiac disease, long history of syncope, syncope after typical triggers even without prodromes, after exercise, after neck compression/rotation, syncope without triggering factors but preceded/followed by nausea and/or vomiting, TLOC in the supine position but preceded by nausea, abdominal pain, or urgency to defecate.

Abbreviations: AF: atrial fibrillation; AV: atrioventricular; BP: blood pressure; ECG: electrocardiogram; ICD: implantable cardiac defibrillator; OH: orthostatic hypotension; PM: pacemaker; QTc: corrected QT interval; TLOC: temporary loss of consciousness; VT: ventricular tachycardia.

**Table 4:** Orthostatic challenge tests.

**Test**	**Method**	**Results**
Active standing test	Measure the BP in clinostatism and after rapid orthostatism at minute 0, minute 1, and minute 3	A symptomatic SBP fall of >20 mmHg or a DBP fall of > 10 mmHg, or absolute an BP value of ≤90 mmHg indicates OH; In reflex OH, the HR increases slightly (<10 bpm); In hypovolemia, the HR increases significantly.
Tilt test	Place the patient in the supine position for at least 5 min (without IVC) or at least 20 min (with IVC); Tilt the table to an angle of 60–70° for 20–45 min (passive phase); If nothing happens during the passive phase, repeat the test after the administration of 300–400 μg of sublingual nitroglycerin (pharmacological phase); Lower the table in less than 15 s; Record a video for additional information if necessary.	Positive if syncope occurs Low specificity and sensitivity in patients with syncope of uncertain cause
Valsalva maneuver	Indicated in patients with suspected reflex syncope or to confirm the tendency of hypotension induced by situational syncope	Results can help diagnose neurogenic syncope or confirm hypotension tendency induced with situational syncope
Deep breathing test	Indicated in patients with OH of suspected neurogenic origin	Results can provide information about reflex syncope
ABPM	Identifies any drops in BP occurring in the 24 h.	Can identify different patterns: dipping (BP falls > 10% at night compared with daytime) or non-dipping (BP falls < 10% at night), or reverse dipping (BP rises at night)

Abbreviations: ABPM: 24-h ambulatory blood pressure monitoring; BP: blood pressure; DBP: diastolic blood pressure; HR: heart rate; IVC: venous cannulation; SBP: systolic blood pressure; TLOC: temporary loss of consciousness.

**Table 5:** Diagnostic tools for cardiac syncope.

**Test**	**Methods**	**Results**
Telemetry ECG monitoring	Used in the acute phase (within 72 h after TLOC) in high-risk patients	Positive if it captures intermittent tachy-or–brady-arrhythmias Low diagnostic power (1.9–17.6%) but justified for high-risk cases
24-h Holter ECG	Indicated in patients with very frequent syncope or presyncope (>1/week)	Positive if it captures relevant arrhythmias; More effective in very frequent syncope cases; Event recorders may be preferred for sporadic episodes.
Event recorder	External Recurrent syncope (>1/month) Internal Indeterminate syncope in high-risk patients; Low-risk patients with a high probability of recurrence within 36 months (≥3 syncopes in the previous 2 years); Identify bradycardia in reflex syncope. Indications Branch block with likely paroxysmal AV block despite a negative EPS; Structural heart disease and/or non-sustained VT with likely arrhythmia despite a negative EPS; Suspected drug-resistant epilepsy; Major depression and frequent unexplained episodes of TLOC; Older patients with unexplained falls; ARVC.	Positive if it captures relevant arrhythmias
EPS	Recommended for: IHD; Sinus bradycardia < 50 bpm; BFB; Syncope with brief palpitations.	In IHD, positive if the induction of sustained monomorphic VT occurs; In sinus bradycardia, if the sinus node recovery time is <1.6 s, PM implantation is indicated; In BFB, PM implantation is indicated if the prolonged HV interval is ≥70 ms or a second-degree AV block is induced during incremental pacing or pharmacological challenge; In syncope with brief palpitations, the finding of sustained or non-sustained VT is diagnostic, and treatment is ablation; A negative EPS does not exclude arrhythmogenic syncope; Not useful if there is a normal ECG and no structural heart disease.
Echocardiography	Useful for diagnosing structural heart disease and assessing the prognosis	EF < 35% indicates a high risk of sudden cardiac death within 2 years; In HCM, LVOTO with a gradient ≥ 50 mmHg is considered significant; Other relevant diagnostic findings include severe AS, cardiac tumors or thrombi, cardiac tamponade, AD, HCM, and congenital CAAs.
Exercise testing	Indicated in patients with syncope on exertion	Positive if syncope is reproduced during or immediately after exertion with associated ECG changes.

Abbreviations: AD: aortic dissection; ARVC: arrhythmogenic right ventricular cardiomyopathy; AS: aortic stenosis; AV: atrioventricular; BFB: bifascicular block; BP: blood pressure; CAAs: coronary artery abnormalities; EF: ejection fraction; EPS: electrophysiological study; HR: heart rate; HCM: hypertrophic cardiomyopathy; IHD: ischemic heart disease; LVOTO: left ventricular outflow tract obstruction; PM: pacemaker; SBP: systolic blood pressure; TLOC: temporary loss of consciousness; VT: ventricular tachycardia.

## Data Availability

No new data were created or analyzed in this study. Data sharing is not applicable to this article.
